# Myocardial salvage by contrast-enhanced cine MR imaging: validation study against conventional T2 edema imaging and angiographic estimates of myocardium at risk during acute myocardial infarction

**DOI:** 10.1186/1532-429X-11-S1-P196

**Published:** 2009-01-28

**Authors:** Ricardo Loureiro, Hiram G Bezerra, Joalbo M Andrade, Chun-Ho Yun, Ammar Sarwar, Michael Bolen, Ricardo C Cury

**Affiliations:** 1 Massachusetts General Hospital – Harvard Medical School, Boston, MA USA; 2 Department of Radiology – Universidade de Brasília, Brasília, Brazil

**Keywords:** Myocardial Mass, Myocardial Salvage, Cine Magnetic Resonance Imaging, Cine Magnetic Resonance, Signal Intensity Difference

## Purpose

To prospectively compare contrast-enhanced (CE) cine steady-state free precession (SSFP) magnetic resonance (MR) imaging to assess area at risk and myocardial salvage with T2-weighted (T2W) edema imaging and the "APPROACH angiographic score", a pathologically-proven angiographic estimate of myocardium at risk, after acute reperfused myocardial infarction (MI).

## Materials and methods

The study was HIPAA compliant and was approved by the institutional review board. All participants gave written informed consent. Twenty one patients (19 men; mean age, 59 ± 12 years) were examined with *T* 2-weighted FSE imaging, CE cineSSFP MR imaging and delayed-enhancement cardiac MR imaging after a first reperfused acute MI. In 15 patients, a second scan was performed 3–6 months later to assess global functional recovery. A modified APPROACH score was calculated from the culprit lesion, location and severity in the angiographic tree.

## Results

Myocardial regions of increased signal intensity were observed in 16 of 21 (76%) examinations on T2W edema imaging and with CE cineSSFP MR imaging at all but one examination (20/21, 95%). CE cineSSFP abnormality was comparable to the size of the hyperintense zone on T2W imaging (20.6 ± 7.0 % versus 19.6 ± 8.1 % of the myocardial mass; *P* = NS) and to the APPROACH risk score (23.2 ± 12.2 % of myocardial mass; P = NS). The infarcted zone was significantly smaller (13.3 ± 7.2; all *P* < 0.05). Myocardial salvage, as measured by the difference of CE cineSSFP MR imaging and delayed-enhancement MRI, showed good correlation with global LV recovery and was comparable to those correlations observed for both T2W edema imaging and APPROACH risk score (r = 0.68 for CEcineSSFP; r = 0.58 for T2W; r = 0.70 for APPROACH). While signal intensity differences between the abnormal and normal myocardium were comparable for T2W imaging and CE cineSSFP sequence, CE cineSSFP MR had a better contrast-to-noise ratio (CNR) and signal-to-noise ratio (SNR) for differentiation between injured and normal regions than did T2W edema imaging (*P* = 0.001 for SNR; P = 0.02 for CNR). The interobserver agreement (Kappa) for CE cineSSFP MR imaging (0.98) was better than that for T2W edema imaging (0.68).

## Conclusion

CE cineSSFP signal abnormality depicts the area at risk in close agreement with the T2W edema imaging and the angiographic risk score, but with superior interobserver agreement and better image quality. The extent of salvaged myocardium, measured as the difference between myocardium at risk and myocardial necrosis, was associated with improvement of LVEF over time.

